# Integrated transcriptomic and metabolomic analyses provide new insights into alkaline stress tolerance in *Gossypium hirsutum*

**DOI:** 10.3389/fpls.2025.1604606

**Published:** 2025-06-03

**Authors:** Shiwei Geng, Wenju Gao, Fenglei Sun, Ni Yang, Teng Ma, Tingwei Wang, Bingyue Wang, Junhao Wang, Shuaishuai Qian, Shengmei Li, Jieyin Zhao

**Affiliations:** ^1^ Xinjiang Cotton Technology Innovation Center/Xinjiang Key Laboratory of Cotton Genetic Improvement and Intelligent Production/National Cotton Engineering Technology Research Center, Cotton Research Institute of Xinjiang Uyghur Autonomous Region Academy of Agricultural Sciences, Wulumuqi, Xinjiang, China; ^2^ National Key Laboratory of Cotton Bio-breeding and Integrated Utilization, Institute of Cotton Research of Chinese Academy of Agricultural Sciences (CAAS), Anyang, China; ^3^ Engineering Research Centre of Cotton, Ministry of Education/College of Agriculture, Xinjiang Agricultural University, Urumqi, China; ^4^ College of Biotechnology, Xinjiang Agricultural Vocational and Technical University, Changji, Xinjiang, China

**Keywords:** *Gossypium hirsutum*, alkaline stress, RNA-seq, metabolome, candidate genes

## Abstract

**Introduction:**

Cotton, one of the most important economic crops worldwide, has long been bred mainly for improvements in yield and quality, with relatively little focus on salt–alkali resistance.

**Methods:**

In this study, transcriptomic and metabolomic sequencing were performed on *Gossypium hirsutum* exposed to alkaline stress for different durations.

**Results:**

The results of sample clustering, principal component analysis (PCA), and the number of differentially expressed genes (DEGs) revealed that 12 hours and 24 hours were the periods during which upland cotton presented the strongest response to salt stress, with flavonoid biosynthesis and alpha-linolenic acid metabolism playing significant roles during this time. A total of 6,610 DEGs were identified via comparison to the 0 h time point, including 579 transcription factors (TFs) that were significantly enriched in pathways such as flavonoid biosynthesis, the cell cycle, the cytochrome P450 pathway, phenylalanine metabolism, phototransduction, and alpha-linolenic acid metabolism. Through ultrahigh-performance liquid chromatography–MS (UPLC-MS), 4,225 metabolites were identified, and 1,684 differentially accumulated metabolites (DAMs) were identified by comparison to the levels at 0 h. A joint analysis of RNA-seq and metabolomic data revealed that the flavonoid biosynthesis and alpha-linolenic acid metabolism pathways play key roles in the response of *G. hirsutum* to alkaline stress, and the key genes in these pathways were identified. The weighted gene correlation network analysis (WGCNA) revealed 15 candidate genes associated with alkali tolerance in cotton, including 4 TFs and 4 genes related to flavonoid and anthocyanin biosynthesis.

**Conclusion:**

In conclusion, our study provides a theoretical foundation for understanding the molecular mechanisms underlying alkali tolerance in cotton and offers new gene resources for future research.

## 1 Introduction

Cotton is an important economic crop worldwide. The focus of cotton breeding has long been on improving yield, quality and stress resistance, while research on alkali resistance has been relatively limited. In recent years, Xinjiang has become China’s largest cotton production base, with the total output accounting for more than 90% of the national total. Xinjiang has a wide area of saline–alkaline land, accounting for approximately one-third of the total land area. Highly alkaline soil tends to shrink, become hard, crack, and compact when it is dry and expands, and becomes muddy and experiences poor aeration when it is wet, making it difficult for cotton to grow normally and seriously affecting yield and fiber quality (Fan et al., 2022). In addition, the widespread distribution of alkaline soil has become an important environmental factor restricting global agricultural production (Litalien and Zeeb, 2020; Rahman et al., 2021).

Compared with salt stress, alkaline stress in high-pH environments is more likely to induce oxidative stress in plants, leading to more severe damage (Cui et al., 2022, 2025). In general, a plant’s alkali tolerance is closely related to the structure of its root system. For example, maize roots possess a hard outer cortex, making maize more alkali tolerant than plants without such a layer (Cao et al., 2022). Cotton, with its well-developed deep root system, can secrete secondary metabolites such as polysaccharides and polyphenols, granting it some alkali tolerance. However, the alkali tolerance of cotton varies according to species, variety, and growth stage. Under alkaline stress, cotton plants experience both osmotic and oxidative stress caused by high ion concentrations, which can result in difficulties during germination, wilting of leaves, and a negative impact on photosynthesis (An et al., 2020). On the other hand, high-pH toxicity also causes significant harm to plants. First, high pH alters the state of minerals in the soil, further affecting the physiological and ecological changes in the plant’s root system. In severe cases, these changes can lead to morphological changes in the roots or even a loss of function. Under acidic conditions, plant cells grow and extend rapidly. However, when the pH of the intercellular medium increases to alkaline levels, the loosening of the cell wall is hindered, impeding cell elongation and inhibiting root hair growth (Liu et al., 2022). Additionally, studies have shown that pH is related to the opening and closing of stomata, with changes in the pH of guard cells occurring during this process (Caine et al., 2023).

With the advancement of sequencing technologies and the reduction in sequencing costs, multiomics approaches have been widely applied in the study of plant growth, development, and stress tolerance (Romero-Losada et al., 2025; Wu et al., 2025; Zhang et al., 2025). Transcriptomics and metabolomics are two crucial high-throughput technologies that play key roles in the study of plant stress tolerance (Du et al., 2025; Lv et al., 2025). Transcriptomics allows an investigation of the expression patterns and regulatory networks of plant genes, whereas metabolomics focuses on the types and quantities of plant metabolites. Together, these two approaches can comprehensively reveal the essential regulatory pathways and candidate genes involved in plant responses to stress conditions. By integrating transcriptomic and metabolomic data, key genes, important metabolic pathways, and regulatory networks associated with stress tolerance can be identified, providing a theoretical basis for breeding stress-resistant varieties and understanding stress tolerance mechanisms (Choudhary et al., 2025). Combined transcriptomic and metabolomic analyses of the salt–alkali-tolerant rapeseed variety SCKY-6–27 revealed that the most highly enriched pathways for the differentially expressed genes (DEGs) and metabolites were starch, sucrose metabolism, and plant hormone signal transduction (Ma et al., 2025). A similar combined analysis of two alfalfa varieties under cold saline–alkali stress revealed significant differences in gene expression and flavonoid contents in the flavonoid biosynthesis pathway, with a further analysis suggesting that the *MsMYB12* gene may respond to stress by regulating the flavonoid biosynthesis pathway (Liu et al., 2024). In a study of two castor bean varieties (ZB8, alkali-sensitive; JX22, alkali-tolerant) under alkaline stress, transcriptomic and metabolomic analyses revealed that alkaline stress induced the upregulation of the *ACX1* and *RBOHD* genes in JX22, increasing reactive oxygen species (ROS) signaling and subsequent stress response regulation. In contrast, ZB8 relies on less efficient nonenzymatic systems, such as carotenoid antioxidants, to mitigate oxidative damage, with genes such as *CCD7* and *CYP897B*, as well as metabolites such as lutein and zeaxanthin, playing crucial roles (Cui et al., 2025). A study of the transcriptional and metabolic responses of wheat root exudates to alkaline stress revealed that the secretion of various metabolites containing –COOH groups is an important regulatory strategy for wheat under alkaline stress. Increased glycolysis, fatty acid synthesis, and phenolic acid synthesis provide additional energy and substrates to help wheat respond to alkaline stress (Wang et al., 2024b).

RNA-seq and metabolomic analyses have revealed numerous key regulatory networks and genes associated with cotton fiber quality and stress tolerance, providing important insights for cotton improvement. However, research on the alkali tolerance of cotton remains relatively limited, with recent studies focused primarily on the fiber quality, drought resistance, salt tolerance, and heat tolerance. Molecular studies of the alkali tolerance mechanisms of *G. hirsutum* are still rare. Therefore, identifying genes related to alkali tolerance in cotton, exploring their regulatory networks and metabolic pathways, and understanding the underlying molecular mechanisms to develop new alkali-tolerant lines and varieties constitute the most cost-effective and efficient approaches. In this study, *G. hirsutum* was subjected to alkaline stress treatments for various durations, and transcriptomic and metabolomic sequencing were performed. DEGs and metabolites were clustered, enriched, and analyzed for transcription factor (TF) expression. Through weighted gene coexpression network analysis (WGCNA) and qRT–PCR, key pathways and genes related to cotton alkali tolerance were identified. These findings provide a theoretical foundation for further research on the molecular mechanisms of alkali tolerance in cotton and new genetic resources for alkali tolerance studies.

## 2 Materials and methods

### 2.1 Plant materials

The cotton material used in this study was the *G. hirsutum* variety Xinlu Zhong 61, which was provided by the Economic Crop Research Institute of the Xinjiang Academy of Agricultural Sciences. Seeds of appropriate maturity, plumpness, and uniformity were selected and sown into pots filled with a mixture of perlite and sterilized soil (1:2 ratio), with four seeds per pot. The daytime temperature in the laboratory was maintained at 25–28°C, with incandescent lighting provided on a 16-h light/8-h dark cycle for germination. After germination, seedlings with consistent growth were selected, and the substrate attached to the roots was gently removed before seedlings were transferred to a hydroponic box for cultivation. The hydroponic box was a 30 L plastic container, and the culture mixture was Hoagland’s nutrient mixture, which was changed weekly. A foam board, cut to fit the culture box, was placed on top of the box, with uniformly spaced holes (2 cm in diameter, 15 holes per board) for plant insertion. The stems were wrapped in sponge strips and inserted into the holes in the foam board, with the roots submerged in the nutrient mixture. Continuous and uniform aeration was provided by an air pump to ensure proper root respiration. When the seedlings reached the three-leaf stage, they were treated with 100 mM NaHCO_3_ to induce alkaline stress. The plants were grown in a growth chamber with a daytime temperature of 28°C, a nighttime temperature of 25°C, and a 16-h light/8-h dark cycle. Samples were collected at 0 h, 2 h, 4 h, 8 h, 12 h, and 24 h of stress treatment, rapidly frozen in liquid nitrogen, and stored at -80°C for subsequent RNA-seq, metabolomic sequencing, and qRT–PCR analyses.

### 2.2 RNA-seq sequencing and analysis

The samples were sent on dry ice to Mavimetabolism (Wuhan, China) for RNA-seq. RNA extraction was performed using TRIzol reagent (Invitrogen). A certain amount of total RNA was extracted and fragmented into smaller pieces. The fragmented mRNA was then mixed with primers, and first-strand cDNA was synthesized using PCR. Next, a second-strand synthesis reaction was performed, and the second-strand products were recovered. The resulting cDNA was subjected to end repair, the addition of an A base, and adapter ligation. PCR amplification of the ligated products was performed, followed by purification and recovery. The final library was tagged, completing cDNA library construction. Library quality was assessed using an Agilent 2100 instrument and Q–PCR. The constructed library was sequenced using the Illumina HiSeq 2500 platform. After the raw sequencing data were obtained, Fastp software (Chen et al., 2018) was used to remove adapter sequences, filter low-quality reads, and eliminate sequences with a greater than 5% N content, resulting in clean reads suitable for analysis. The clean reads were aligned to the *G. hirsutum* TM-1 reference genome (https://www.cottongen.org/species/Gossypium_hirsutum/ZJU-AD1_v2.1) using HISAT2 (Pertea et al., 2016). The alignment results were quantified using featureCounts. Setting the FDR standard to less than 0.01 ensures that fewer than 1% of the DEGs screened are caused by random differences. FDR<0.01 and |log_2_fold change|>1 were used as the criteria for screening DEGs (Love et al., 2014). GO and KEGG enrichment analyses were performed on all DEGs using the clusterProfiler (version 4.14.4) package (Wu et al., 2021), with statistical significance determined by hypergeometric tests. The protein sequences of all DEGs were submitted to PlantTFDB (https://planttfdb.gao-lab.org) for analysis and prediction to obtain differentially expressed transcription factors (TFs).

### 2.3 Metabolite extraction

After vacuum freeze-drying, 50 mg of the sample was weighed, mixed with 1000 μL of extraction solution (methanol/acetonitrile/water, 2:2:1 v/v) and vortexed for 30 seconds. Steel beads were added, and the sample was processed with a 45 Hz grinder for 10 minutes, followed by ultrasonication for 10 minutes (in an ice–water bath). The sample was then left to stand at -20°C for 1 h, followed by centrifugation at 12,000 rpm for 15 minutes. The supernatant (500 μL) was carefully collected and transferred to an EP tube. The extract was dried in a vacuum concentrator and then redissolved in 160 μL of extraction solution (acetonitrile/water, 1:1, v/v). The mixture was vortexed for 30 seconds, ultrasonicated in an ice–water bath for 10 minutes, and centrifuged again at 12,000 rpm for 15 minutes. The supernatant was carefully collected for subsequent analysis. Metabolites were analyzed using a Waters Acquity I-Class PLUS ultrahigh-performance liquid chromatography system coupled with an AB Sciex Qtrap 6500+ high-sensitivity mass spectrometer. Chromatographic separation was performed on a Waters Acquity UPLC HSS T3 column (1.8 μm, 2.1*100 mm) with an injection volume of 2 μL. The mass spectrometry conditions were as follows: electrospray ionization (ESI) temperature of 550°C; ion spray voltage (IS) of 5,500 V in positive ion mode and -4,500 V in negative ion mode; and ion source gases I (GSI), II (GSII), and curtain gas (CUR) set to 50 psi, 55 psi, and 35 psi, respectively, with collision-induced dissociation (CID) parameters set to moderate intensity.

### 2.4 Metabolomic analysis

Metabolite identification was performed with the in-house database GB-PLANT using secondary mass spectrometry data. Isotope signals, redundant signals from K^+^, Na^+^, and NH_4_^+^ ions, and fragment ions corresponding to higher-molecular-weight compounds were removed. The metabolites were quantified in multiple reaction monitoring (MRM) mode with a triple quadrupole mass spectrometer. After the mass spectrometry data for different samples were obtained, the peak areas of all the metabolites were corrected. Principal component analysis (PCA) was conducted on the metabolite content data matrix using R software. Metabolite classification and pathway functional annotations were performed using the KEGG database (http://www.genome.ad.jp/kegg/) to identify the major biochemical metabolic and signal transduction pathways involved. Partial least squares regression (PLSR) was applied to establish a model of the relationships between metabolite levels and sample categories for predictive modeling. DAMs were identified using the criteria of a fold change >2 or a fold change < 1/2 and a P value <0.05.

### 2.5 WGCNA

The gene expression profiles of the DEGs were subjected to a coexpression analysis using the dynamic branch cutting method in the R package WGCNA (Langfelder and Horvath, 2008). The weighting coefficient β was chosen to yield a correlation coefficient of approximately 0.8 with a certain level of gene connectivity to ensure a scale-free network. In this study, β=8 was selected as the weighting coefficient. The network was constructed via an automatic network construction function, blockwise modules, resulting in multiple valid modules, each containing a different number of genes. Modules with a similarity greater than 0.75 were merged using minModuleSize = 30 and Merge Cut Height = 0.25 as the criteria. The module eigengene (ME) was calculated and correlated with hormone levels and different treatment durations. Specific modules were selected using the criteria of r>0.80 and P<0.05. The coexpression network was visualized using Cytoscape (Shannon et al., 2003) (version 3.10.0) software.

### 2.6 qRT–PCR

Homologs of the candidate genes were identified using the BLASTn (Altschul et al., 1990) function on the CottonGen (Yu et al., 2021) website (https://www.cottongen.org/), and specific primers were designed using DNAMan (Lynnon Corporation, Canada). Total RNA was extracted from samples collected after various durations of stress with the RNAprep Pure Polysaccharide Polyphenol Plant Total RNA Extraction Kit (Tiangen, Beijing, China) according to the manufacturer’s instructions, and first-strand cDNA was synthesized using a reverse transcription kit (abm). Cotton *Ubiquitin7* (*GhUBQ7*) was selected as the internal reference gene, and qRT–PCR amplification of the relevant genes was performed on an Applied Biosystems™ 7500 Fast Real-Time PCR System (three biological replicates). Relative gene expression was analyzed using the 2^−ΔΔCt^ method (Livak and Schmittgen, 2001), and the data were visualized using GraphPad Prism version 8.0.1 for Windows. All primers used in this study are listed in **Supplementary Table S2**.

## 3 Results

### 3.1 Overall analysis of RNA-seq data

**Figure 1:**
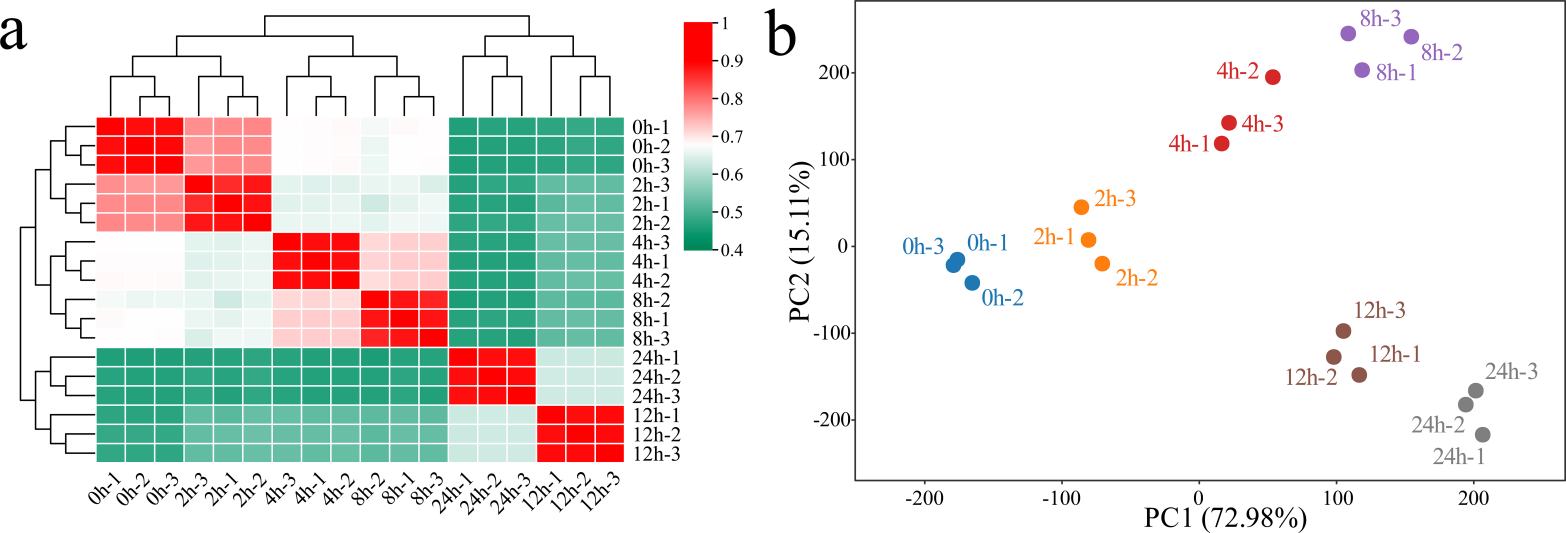
Correlation analysis and PCA of the RNA-seq data from 18 (*G*) *hirsutum* samples under alkaline stress. **(a)** Correlation analysis of the samples; a value from 0.4 to 1 represents the magnitude of the correlation coefficient between samples, with 0.4 indicating the lowest correlation coefficient and 1 indicating the highest correlation coefficient between samples. **(b)** PCA of the samples; each point represents a sample, with different colors used to identify processing at different times.

RNA-seq was performed on 18 samples of *G. hirsutum* subjected to alkaline stress at six time points (0 h, 2 h, 4 h, 8 h, 12 h, and 24 h). After filtering, we obtained a total of 153.89 Gb of clean data, with each sample yielding more than 6.65 Gb of clean data. The Q30 base percentage exceeded 95.90%, and the GC content was above 43.55% (**Supplementary Table S1**). The correlation between biological replicates not only indicates the reproducibility of experimental procedures but also reflects the reliability of the DEG identification and helps identify potential outliers. The Pearson correlation coefficients for the three biological replicates of the same sample were greater than 0.96 (**Figure 1a**). Principal component analysis (PCA) revealed that samples from the same biological replicate clustered together, confirming the reliability and reproducibility of the RNA-seq data (**Figure 1b**). PCA revealed a significant separation trend among the samples from the alkaline stress control group and groups exposed to alkaline stress for different times. As the duration of stress increased, the distance between the samples from the treatment groups and the control samples gradually increased, suggesting that the response of *G. hirsutum* to alkaline stress became more pronounced with prolonged treatment time.

### 3.2 Differential expression analysis

**Figure 2:**
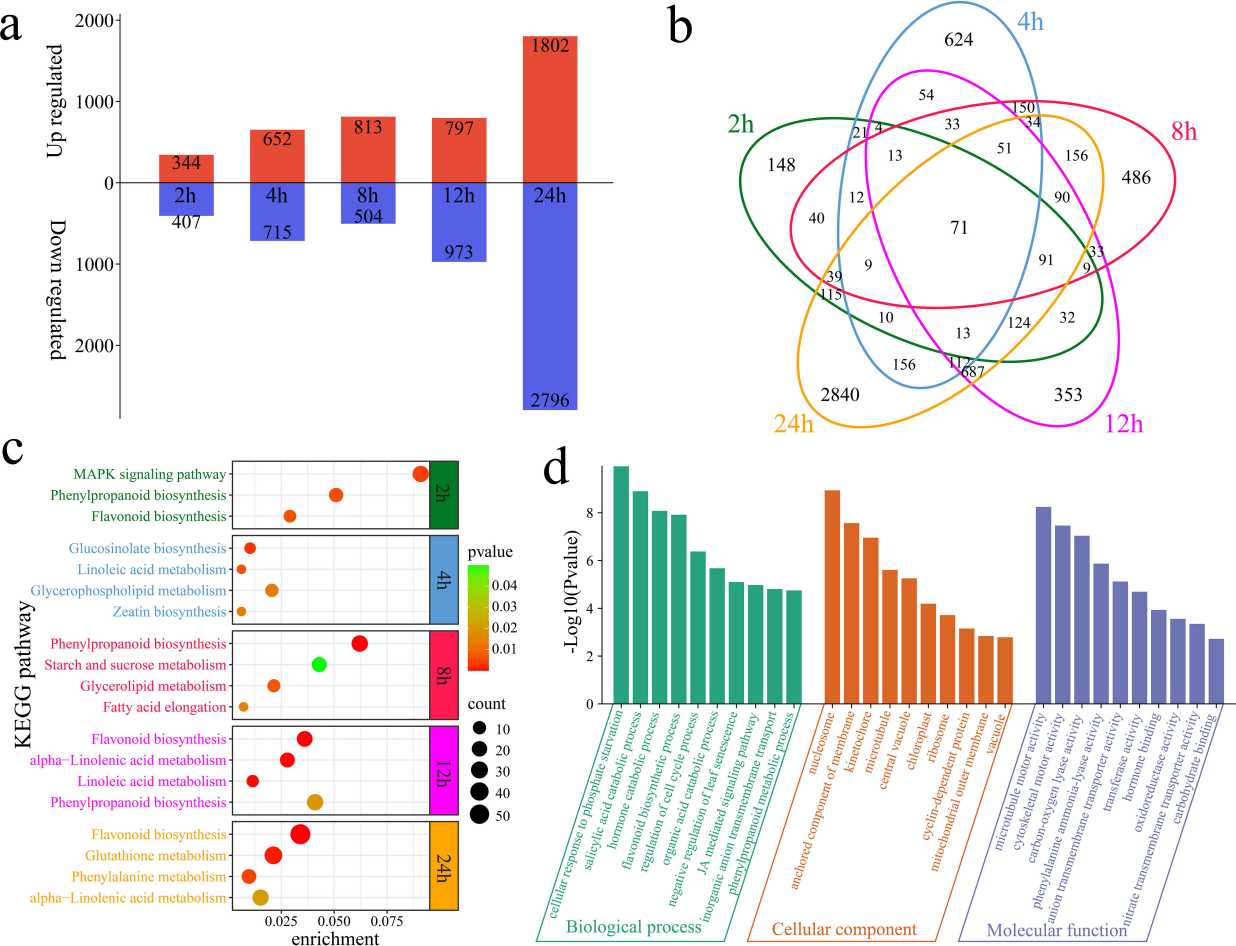
Numbers of DEGs and enrichment analysis results compared with those at 0 h of alkaline stress. **(a)** Numbers of upregulated and downregulated DEGs identified at each time point compared with 0 h of stress. **(b)** Venn diagram of unique and common DEGs identified at each time point compared with 0 h of alkaline stress. **(c)** KEGG enrichment analysis of DEGs identified at various time points compared with 0 h of alkaline stress. **(d)** GO enrichment analysis of all DEGs compared with the genes detected at 0 h of alkaline stress.

The differential expression analysis revealed a total of 751 DEGs at 2 h of alkaline stress compared with 0 h, including 344 upregulated and 407 downregulated genes, with 148 unique DEGs (**Figures 2a, b**). At 4 h of alkaline stress, 1,367 DEGs were identified, with 652 upregulated and 715 downregulated genes and 624 unique DEGs, compared with those at 0 h. At 8 h of alkaline stress, 1,317 DEGs were identified, with 813 upregulated and 504 downregulated genes and 486 unique DEGs, compared with those at 0 h. At 12 h of alkaline stress, 1,770 DEGs were identified, with 797 upregulated and 973 downregulated genes and 353 unique DEGs, compared with those at 0 h. At 24 h of alkaline stress, 4,598 DEGs were identified, with 1,802 upregulated and 2,796 downregulated genes and 2,840 unique DEGs, compared with those at 0 h. In total, 6,610 DEGs were identified in plants under alkaline stress, with 71 common DEGs detected among the groups. KEGG enrichment analyses were conducted for the DEGs at different time points of alkaline stress to reveal the dynamic changes in the response of *G. hirsutum* to alkaline stress (**Figure 2c**). The DEGs at 2 h were significantly annotated to the MAPK signaling pathway, phenylpropanoid biosynthesis pathway and flavonoid biosynthesis pathway. The DEGs at 4 h were significantly annotated to glucosinolate biosynthesis, linoleic acid metabolism, glycerophospholipid metabolism and zeatin biosynthesis pathways. The DEGs at 8 h were significantly annotated to the phenylpropanoid biosynthesis, starch and sucrose metabolism, glycerolipid metabolism and fatty acid elongation pathways. The DEGs at 12 h were significantly annotated to the flavonoid biosynthesis, alpha-linolenic acid metabolism, linoleic acid metabolism and phenylpropanoid biosynthesis pathways. The DEGs at 24 h were significantly annotated to the flavonoid biosynthesis, glutathione metabolism, phenylalanine metabolism and alpha-linolenic acid metabolism pathways. Flavonoid biosynthesis and alpha-linolenic acid metabolism pathways were significantly annotated at 12 h and 24 h, during which the greatest number of DEGs was observed, indicating that these time points are the most intense periods of the response of *G. hirsutum* to alkaline stress and that flavonoid biosynthesis and alpha-linolenic acid metabolism play important roles during these periods. GO enrichment analyses were performed on the 6,610 DEGs. The GO analysis revealed significantly enriched biological processes, including the cellular response to phosphate starvation, the salicylic acid catabolic process, the hormone catabolic process, the flavonoid biosynthetic process, the regulation of the cell cycle process, the organic acid catabolic process, the negative regulation of leaf senescence, the jasmonic acid-mediated signaling pathway, inorganic anion transmembrane transport, and the phenylpropanoid metabolic process (**Figure 2d**).

### 3.3 Clustering analysis of DEGs

**Figure 3:**
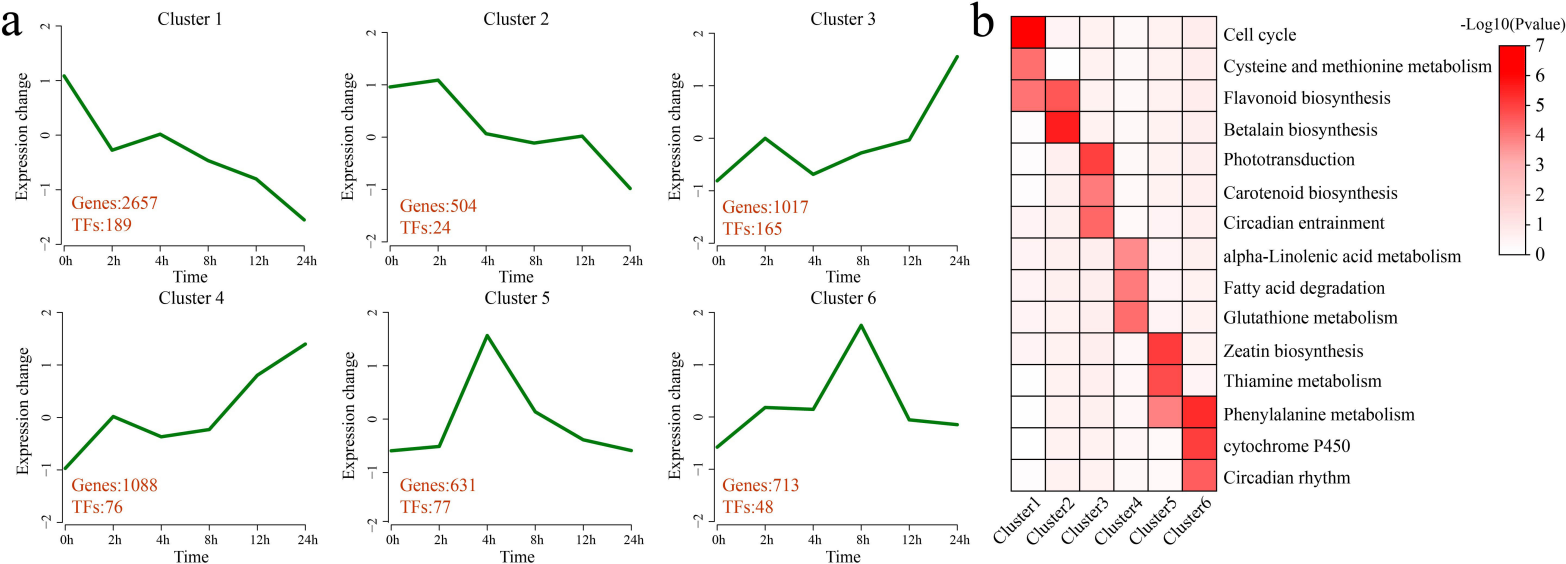
Clustering and enrichment analyses of all DEGs**. (a)** Line plot of the expression patterns of all the DEGs based on the clustering analysis. The red numbers represent the quantity of DEGs and differentially expressed TFs identified in each cluster. **(b)** KEGG pathway enrichment analysis of each category of DEGs. The intensity of the color represents the magnitude of the p value, specifically the value of -log_10_ (p value). A darker color indicates a smaller p value, which corresponds to a larger value of -log_10_ (p value), whereas a lighter color indicates a larger p value, equating to a smaller value of -log_10_ (p value).

K-means clustering was applied to the 6,610 DEGs, identifying 6 statistically significant clusters. A KEGG pathway enrichment analysis was performed for each cluster (**Figures 3a, b**). Cluster 1 presented a decrease in expression at 2 h of stress, an increase at 4 h, and a gradual decrease thereafter, with the lowest expression observed at 24 h. This cluster contained 2,657 DEGs and 189 TFs. Significant enrichment was detected in the following pathways: the cell cycle, cysteine and methionine metabolism, and flavonoid biosynthesis. Cluster 2 showed a gradual decrease in expression after 4 h of stress, with the lowest expression observed at 24 h. This cluster contained 504 DEGs and 24 TFs. Significant enrichment was detected in the flavonoid biosynthesis and betalain biosynthesis pathways. Cluster 3 presented an increase in expression at 2 h of stress, followed by a decrease at 4 h and then a gradual increase, with expression peaking at 24 h. This cluster contained 1,017 DEGs and 165 TFs. Significant enrichment was detected in the phototransduction, carotenoid biosynthesis, and circadian entrainment pathways. Cluster 4 presented an increase in expression at 2 h of stress and a slight decrease from 4 h to 8 h, followed by a gradual increase, with the highest expression occurring at 24 h. This cluster contained 1,088 DEGs and 76 TFs. Significant enrichment was detected in the pathways of alpha-linolenic acid metabolism, fatty acid degradation, and glutathione metabolism. Cluster 5 showed a rapid increase in expression under stress, with expression peaking at 4 h, followed by a gradual decrease. This cluster contained 631 DEGs and 77 TFs. Significant enrichment was detected in the zeatin biosynthesis, thiamine metabolism, and phenylalanine metabolism pathways. Cluster 6 presented an increase in expression under stress, with expression peaking at 8 h, followed by a gradual decrease. This cluster contained 713 DEGs and 48 TFs. Significant enrichment was detected in the phenylalanine metabolism, cytochrome P450, and circadian rhythm pathways.

### 3.4 Metabolomic analysis

**Figure 4:**
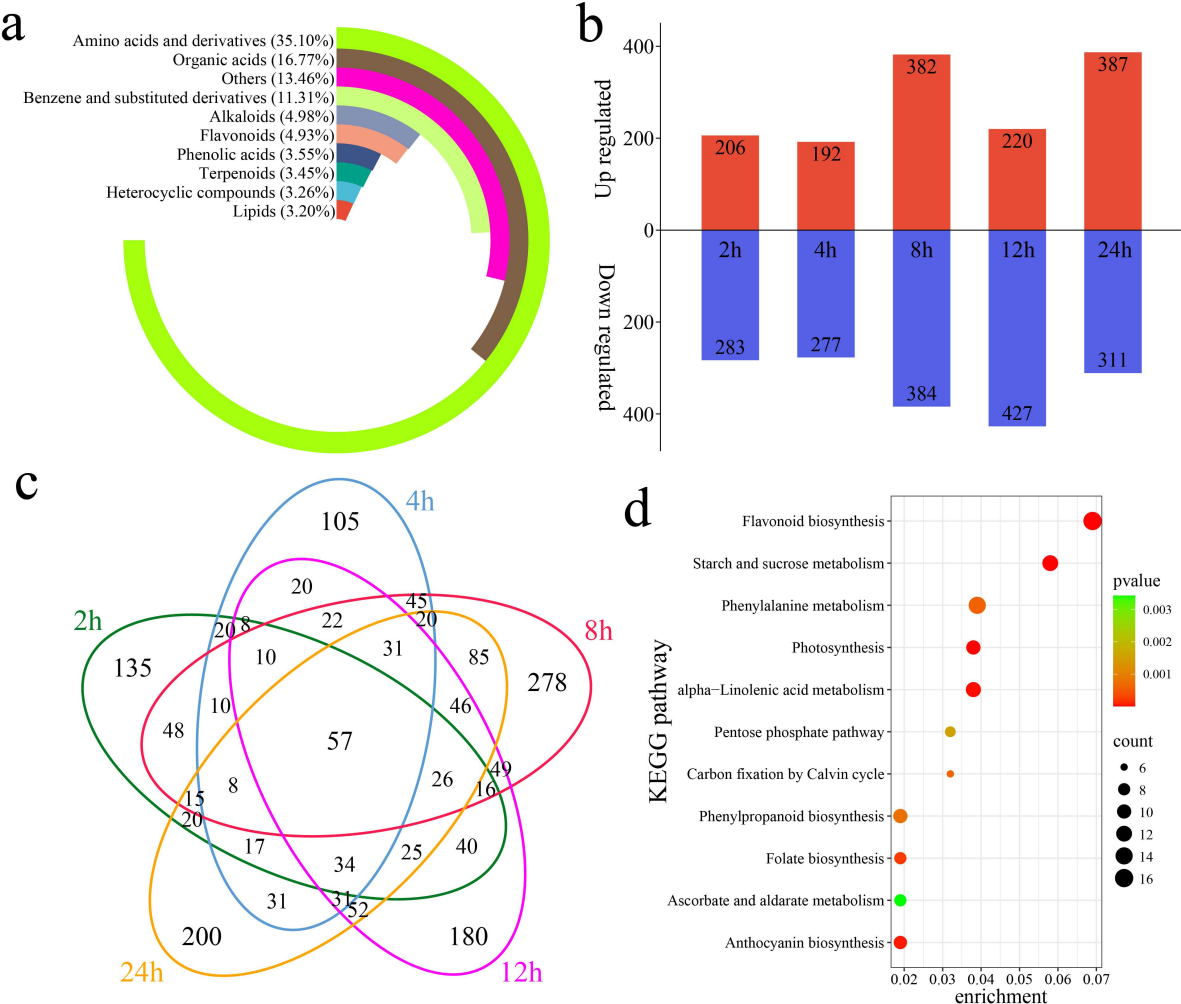
DAMs and enrichment compared with the metabolites detected at 0 h of stress. **(a)** Percentages of classified metabolites, **(b)** numbers of upregulated and downregulated DAMs at each time point compared with that at 0 h of stress, **(c)** Venn diagram showing unique and shared DAMs at each time point compared with those at 0 h of stress, and **(d)** KEGG enrichment analysis of all DAMs compared with the metabolites detected at 0 h of stress.

A total of 4,225 metabolites were identified in 18 samples of *G. hirsutum* subjected to alkaline stress at six time points (0 h, 2 h, 4 h, 8 h, 12 h, and 24 h) via UPLC–MS. PCA revealed that samples of the same biological replicates clustered together, indicating that the metabolomic data were reliable and reproducible (**Supplementary Figure S1**). The 4,225 identified metabolites were classified into 10 categories. Amino acids and their derivatives accounted for 35.10% of the total, organic acids accounted for 16.77%, and benzene and substituted benzene derivatives accounted for 11.31% (**Figure 4a**). Through a differential abundance analysis, a total of 489 differentially accumulated metabolites (DAMs) were identified at 2 h compared with 0 h, with 206 upregulated and 283 downregulated DAMs, including 135 unique DAMs (**Figures 4b, c**). At 4 h, 469 DAMs were identified, with 192 upregulated and 277 downregulated DAMs, including 105 unique DAMs. At 8 h, 766 DAMs were identified, with 382 upregulated and 384 downregulated DAMs, including 278 unique DAMs. At 12 h, 647 DAMs were identified, with 220 upregulated and 427 downregulated DAMs, including 180 unique DAMs. At 24 h, 698 DAMs were identified, with 387 upregulated and 311 downregulated DAMs, including 200 unique DAMs. A total of 1,684 DAMs were identified, including 57 shared DAMs. The KEGG enrichment analysis of the 1,684 DAMs revealed significant enrichment in pathways related to flavonoid biosynthesis, starch and sucrose metabolism, photosynthesis, alpha-linolenic acid metabolism, anthocyanin biosynthesis, folate biosynthesis, phenylalanine metabolism, phenylpropanoid biosynthesis, carbon fixation via the Calvin cycle, the pentose phosphate pathway, and ascorbate and aldarate metabolism (**Figure 4d**).

### 3.5 DAM clustering analysis

**Figure 5:**
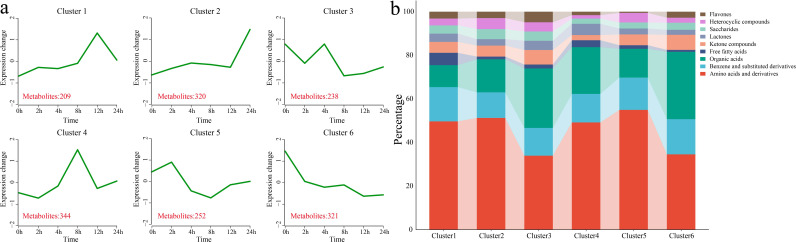
Clustering and classification of all the DAMs. **(a)** Line plot showing the pattern of changes in the contents of all the DAMs based on the clustering analysis; the red numbers represent the quantity of DAMs identified in each cluster. **(b)** Classification of all the DAMs into different categories.

Using k-means clustering, 6 statistically significant clusters were identified from 1,684 DAMs, and metabolic classification was performed for each cluster (**Figures 5a, b**). Cluster 1 showed a gradual increase in metabolite levels under stress, with metabolite levels peaking at 12 h, followed by a decrease. This cluster contained 209 DAMs, classified mainly as amino acids and their derivatives and benzene and substituted benzene derivatives. Cluster 2 maintained a relatively stable metabolite level before 12 h, and the metabolite level increased sharply at 24 h. This cluster contained 320 DAMs, classified mainly as amino acids and their derivatives and benzene and substituted benzene derivatives. Cluster 3 showed a decrease in metabolite levels at 2 h and 8 h, with increases at 4 h and 12 h. This cluster contained 238 DAMs, classified mainly as amino acids and their derivatives, and organic acids. Cluster 4 showed a gradual increase in metabolite levels under stress, with the levels peaking at 8 h, followed by a decrease. This cluster contained 344 DAMs, classified mainly as amino acids and their derivatives and benzene and substituted benzene derivatives. Cluster 5 presented a slight increase in metabolite levels after 2 h of stress, followed by a gradual decrease. This cluster contained 252 DAMs, classified mainly as amino acids and their derivatives, benzene and substituted benzene derivatives, and organic acids. Cluster 6 showed a gradual decrease in metabolite levels under stress, with the minimum levels observed at 24 h. This cluster contained 321 DAMs, classified mainly as amino acids and their derivatives and organic acids.

### 3.6 Combined RNA-seq and metabolomic analyses

**Figure 6:**
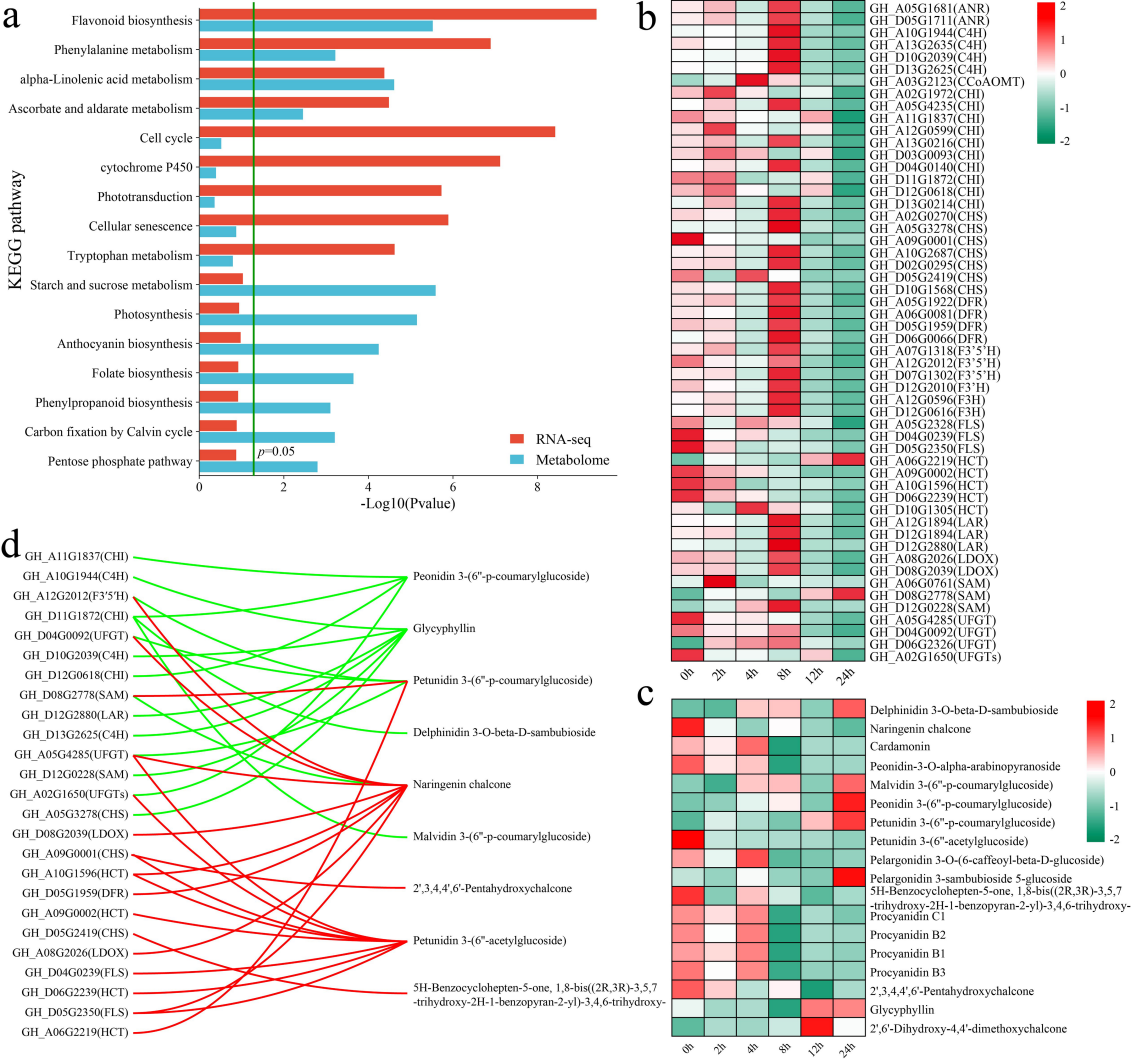
Results of the combined RNA-seq and metabolomic analyses, analysis of changes in DAMs and DEGs in the flavonoid biosynthesis pathway, and correlation analysis. **(a)** KEGG pathway annotations of DAMs and DEGs. **(b)** Changes in the expression of DEGs in the flavonoid biosynthesis pathway were quantified via standardized scoring, with values standardized to range from -2 to 2. **(c)** Heatmap of changes in the levels of DAMs in the flavonoid biosynthesis pathway. Standardized scoring was used, with values standardized to range from -2 to 2. **(d)** Correlation network of flavonoid biosynthesis pathway metabolites and genes; the red line represents a significant positive correlation, and the green line represents a significant negative correlation.

The KEGG enrichment analysis of all the DAMs and DEGs revealed that both groups were significantly enriched in the flavonoid biosynthesis, phenylalanine metabolism, alpha-linolenic acid metabolism, and ascorbate and aldarate metabolism pathways (**Figure 6a**). First, we analyzed the expression patterns of genes in the flavonoid biosynthesis pathway. In addition to the flavonol synthase (FLS) and hydroxycinnamoyl transferase (HCT) genes, other flavonoid biosynthesis genes, such as chalcone isomerase (CHI), chalcone synthase (CHS) and dihydroflavonol 4-reductase (DFR), presented the highest expression at 8 h (**Figure 6b**). Moreover, the levels of flavonoid metabolites, such as delphinidin 3-O-beta-D-sambubioside, malvidin 3-(6’’-p-coumarylglucoside), peonidin 3-(6’’-p-coumarylglucoside), petunidin 3-(6’’-p-coumarylglucoside), pelargonidin 3-sambubioside 5-glucoside, and glycyphyllin, peaked at 24 h (**Figure 6c**). On the other hand, the levels of naringenin chalcone, peonidin-3-O-alpha-arabinopyranoside, petunidin 3-(6’’-acetylglucoside), 5H-benzylohepten-5-one, and 1,8-bis((2R,3R)-3,5,7-trihydroxy-2H-1-benzopyran-2-yl)-3,4,6-trihydroxy, and 2’,3,4,4’,6’-pentahydroxychalcone peaked at 0 h, with their levels decreasing after exposure to stress. We calculated the correlation between genes and metabolites to further explore the relationships between the expression of flavonoid biosynthesis genes and metabolite levels, visualizing those with an absolute correlation coefficient greater than 0.8 and a p value less than 0.05 (**Figure 6d**). A total of 25 flavonoid biosynthesis genes were significantly correlated with 9 flavonoid pathway metabolites, with the expression of 14 genes exhibiting negative correlations with the levels of 6 metabolites and the expression of 16 genes showing positive correlations with the levels of 5 metabolites. Specifically, *GH_D11G1872* (CHI) expression was significantly negatively correlated with the peonidin 3-(6’’-p-coumarylglucoside), delphinidin 3-O-beta-D-sambubioside, and malvidin 3-(6’’-p-coumarylglucoside) levels.

**Figure 7:**
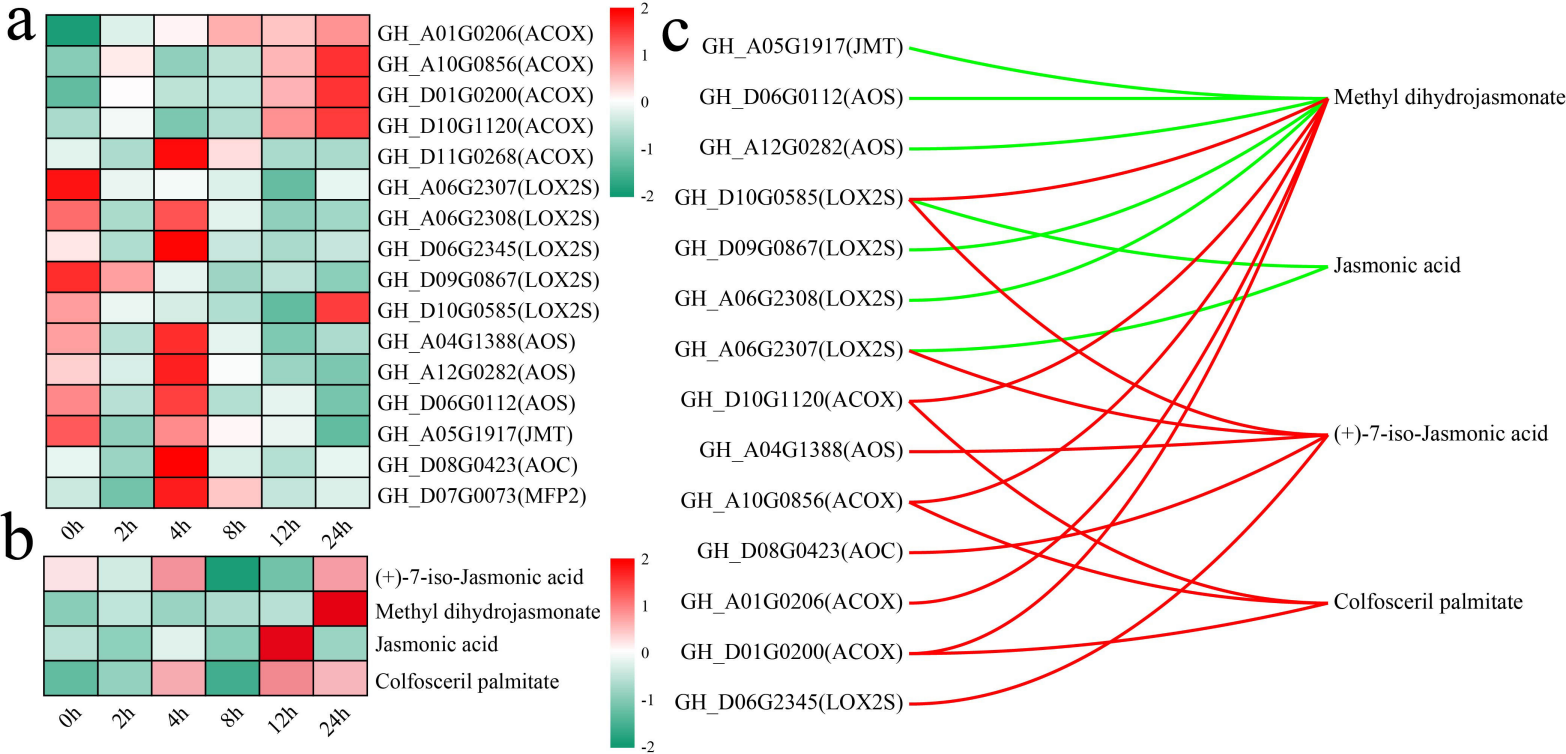
Changes in DAMs and DEGs in the JA biosynthesis pathway and correlation analysis. **(a)** Changes in the expression of DEGs in the JA biosynthesis pathway were quantified via standardized scoring, with values standardized to range from -2 to 2. **(b)** Heatmap of changes in the levels of DAMs in the JA biosynthesis pathway; standardized scoring was used, with values standardized to range from -2 to 2. **(c)** Correlation network between metabolites and genes in the JA biosynthesis pathway; the red line represents a significant positive correlation, and the green line represents a significant negative correlation.

The alpha-linolenic acid metabolism pathway is an important route for the synthesis of jasmonic acid (JA) in plants (Choudhary et al., 2025). Both the alpha-linolenic acid metabolism and JA-mediated signaling pathways were significantly enriched in differentially expressed genes (DEGs) and differentially accumulated metabolites (DAMs) at multiple stages. To this end, an analysis was conducted on the changes in the levels of genes and metabolites associated with other JA biosynthesis pathways, with an initial focus on the variations in the expression levels of JA biosynthesis genes (**Figure 7a**). The rate-limiting enzymes of JA synthesis, allene oxide cyclase (AOC, *GH_D08G0423*) and allene oxide synthase (AOS, *GH_A04G1388*, *GH_A12G0282*, and *GH_D06G0112*), presented the highest expression after 4 h of stress. The JA level peaked at 12 h of stress, whereas the level of methyl dihydrojasmonate peaked at 24 h of stress (**Figure 7b**). The correlations between genes and metabolites were calculated to further explore the relationships between the expression of JA biosynthetic genes and metabolite levels, and those with absolute correlation coefficients greater than 0.8 and p values less than 0.05 were visualized (**Figure 7c**). A total of 14 genes were significantly correlated with 4 metabolites in the JA biosynthesis pathway, with the expression of 7 genes exhibiting significant negative correlations with JA and methyl dihydrojasmonate levels and the expression of 9 genes exhibiting significant positive correlations with methyl dihydrojasmonate, (+)-7-iso-JA, and colfosceril palmitate levels. *GH_D10G0585* (LOX2S) expression was significantly positively correlated with methyl dihydrojasmonate and (+)-7-iso-jasmonic acid levels and significantly negatively correlated with JA levels.

### 3.7 Analysis of differentially expressed TFs

**Figure 8:**
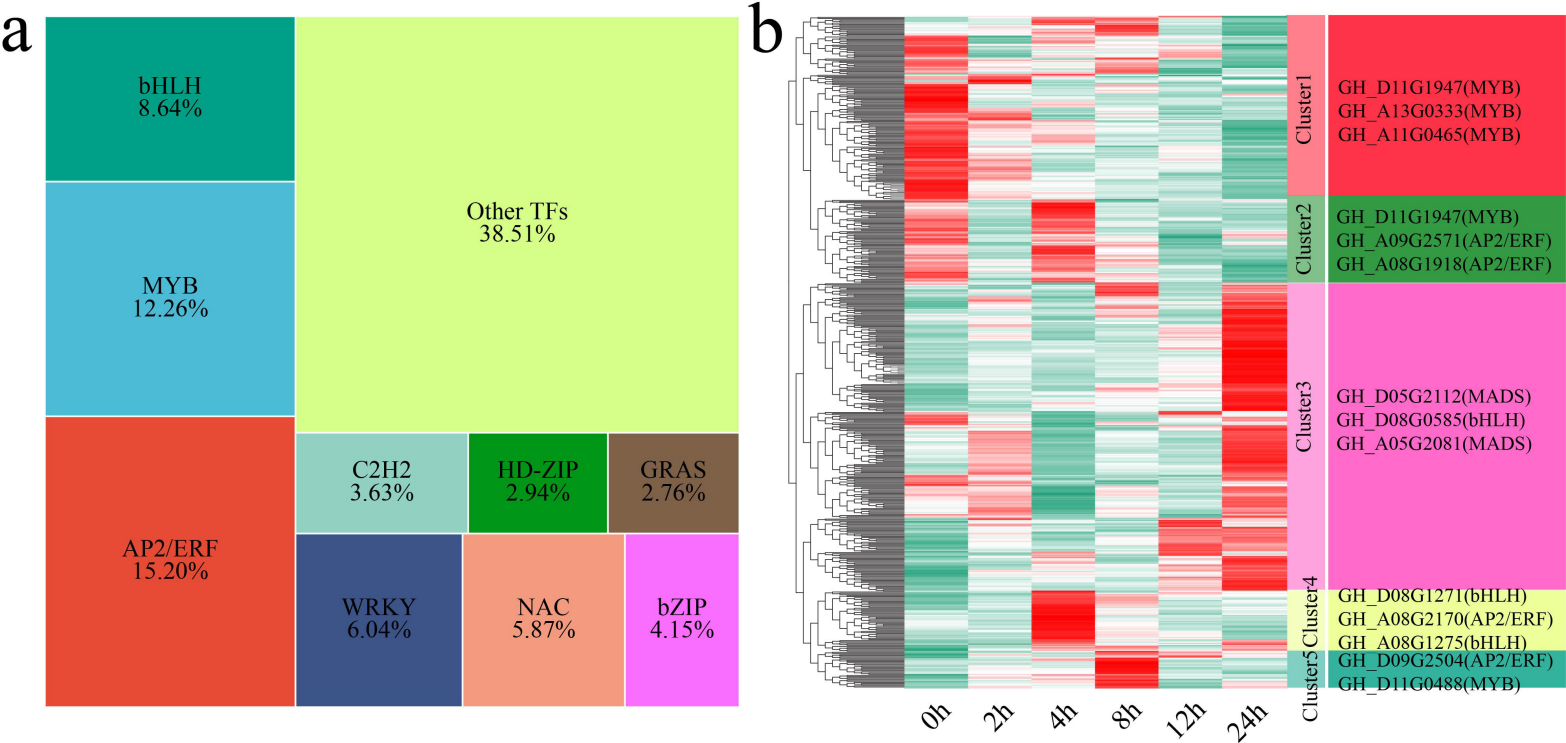
Proportions and expression patterns of differentially expressed TFs. **(a)** Proportional area chart of differentially expressed TFs. **(b)** Heatmap of the differential expression patterns of TFs, with the TFs exhibiting the greatest fold changes shown on the right.

TFs are key regulators of gene expression and play important roles in plant growth, development, and stress response mechanisms (Yan et al., 2025; Yang et al., 2025). In this study, we identified a total of 579 differentially expressed TFs (DE-TFs) from the 6,610 DEGs. The major TF families identified included the AP2/ERF, MYB, bHLH, WRKY, NAC, bZIP, C2H2, HD-ZIP, and GRAS families (**Figure 8a**). Using k-means clustering, we identified 5 statistically significant clusters of these DE-TFs, and we further examined the TFs with the highest fold changes in each cluster (**Figure 8b**). Cluster 1 showed a gradual decrease in TF expression under stress, with the lowest expression occurring at 24 h. The TFs with the greatest fold changes in expression in this cluster were *GH_D11G1947* (MYB), *GH_A13G0333* (MYB) and *GH_A11G0465* (MYB). Cluster 2 presented the highest TF expression at 0 h and 4 h. The TFs with the greatest fold changes in expression in this cluster were *GH_D11G1947* (MYB), *GH_A09G2571* (AP2/ERF) and *GH_A08G1918* (AP2/ERF). Cluster 3 showed an increase in TF expression, with the expression peaking at 24 h. The TFs with the greatest fold changes in expression in this cluster were *GH_D05G2112* (MADS), *GH_D08G0585* (bHLH) and *GH_A05G2081* (MADS). Cluster 4 presented the highest TF expression at 4 h. The TFs with the greatest fold changes in expression in this cluster were *GH_D08G1271* (bHLH), *GH_A08G2170* (AP2/ERF) and *GH_A08G1275* (bHLH). Cluster 5 presented the highest TF expression at 8 h. The TFs with the greatest fold changes in expression in this cluster were *GH_D09G2504* (AP2/ERF) and *GH_D11G0488* (MYB).

### 3.8 WGCNA

**Figure 9:**
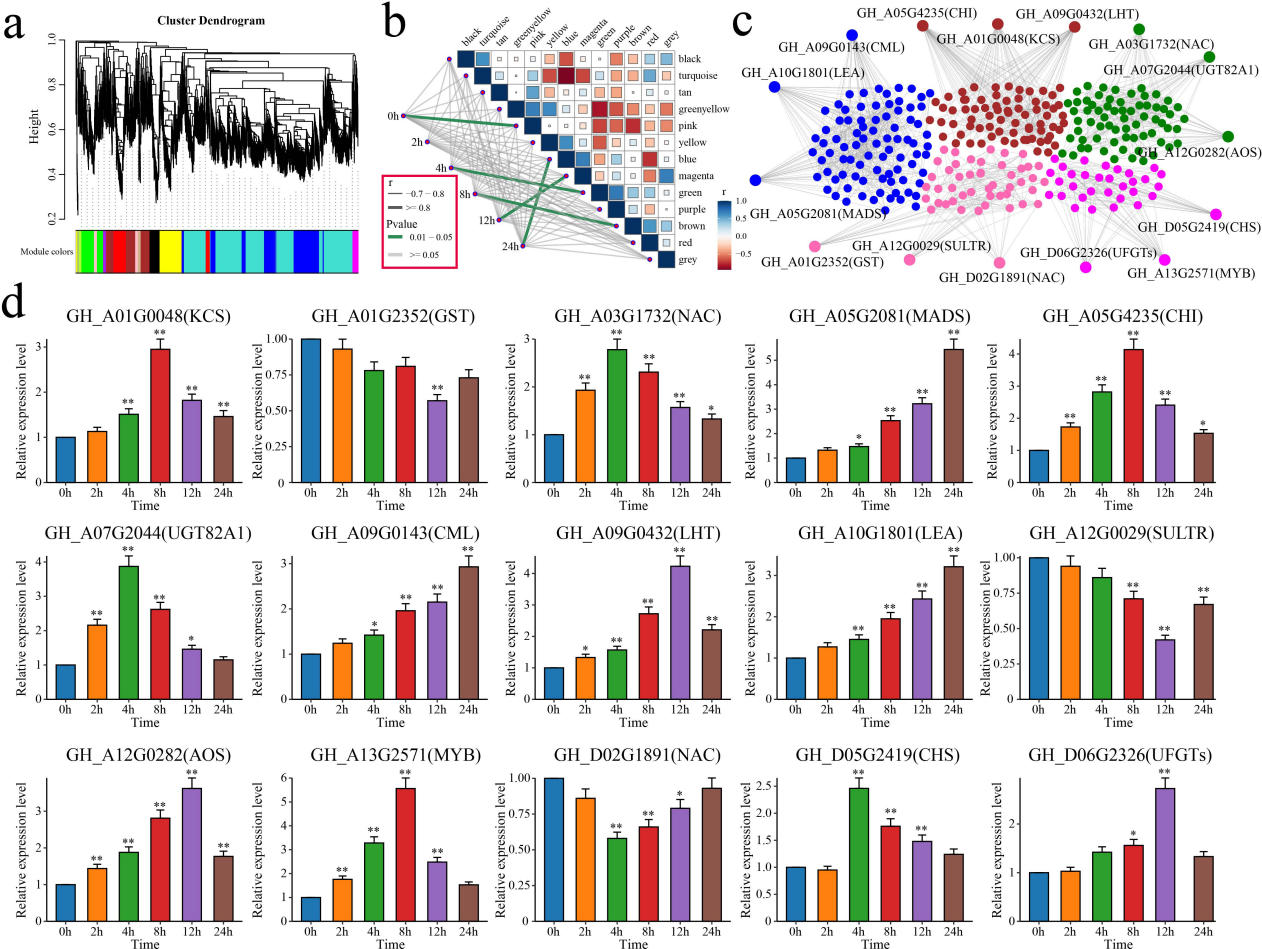
WGCNA and analysis of the candidate gene expression patterns. **(a)** WGCNA clustering dendrogram, with different colors representing different modules. **(b)** Correlation and significance analyses between modules and alkaline stress in cotton at different time points. The thickness of the lines represents the magnitude of the correlation coefficient between the modules and periods, with gray indicating a p value greater than or equal to 0.05 and green indicating a p value less than 0.05. **(c)** Gene network diagrams for the pink, green, brown, magenta, and blue modules. **(d)** Analysis of the expression patterns of the 15 candidate genes in cotton under alkaline stress. The error bars represent the average values ± SDs from three replicates (*P<0.05 and **P<0.01).

WGCNA was performed on the expression matrix of the 6,610 DEGs to identify the core genes related to alkali tolerance in cotton, which resulted in 13 distinct coexpression modules (**Figure 9a**). The correlation between each module and the duration of stress was calculated. The results indicated that the pink module was highly correlated with 0 h, the green module was correlated with 4 h, the brown module was correlated with 8 h, the magenta module was correlated with 12 h, and the blue module was correlated with 24 h (**Figure 9b**). By calculating the kME (eigengene connectivity) value of each module gene, the gene with the highest absolute kME value was used as the hub gene of each module. Three hub genes were identified for each module, resulting in a total of 15 candidate genes associated with salt tolerance in cotton (**Figure 9c**).

The functions of these 15 candidate genes were annotated based on the homologous genes in *A. thaliana* (**Table 1**). *GH_A03G1732* (NAC), *GH_A05G2081* (MADS), *GH_A13G2571* (MYB), and *GH_D02G1891* (NAC) encode four TFs that are involved primarily in the response to stress. *GH_A10G1801* encodes a late embryogenesis abundant (LEA) protein that is crucial for maintaining osmotic pressure and protecting cell membrane structures. *GH_A01G0048* encodes a 3-ketoacyl-CoA synthase (KCS) that is involved in the biosynthesis of very-long-chain fatty acids. *GH_A01G2352* encodes a glutathione S-transferase (GST) that is involved in scavenging ROS. *GH_A05G4235* and *GH_D05G2419* encode a chalcone isomerase (CHI) and a chalcone synthase (CHS), respectively, which are involved in flavonoid biosynthesis. *GH_A07G2044* encodes the UDP-glycosyltransferase 82A1 (UGT82A1), which catalyzes the glycosylation of flavonoids. *GH_A09G0143* encodes a calmodulin-like (CML) protein, a calcium signal effector. *GH_A09G0432* encodes a lysine histidine transporter (LHT) that is involved in amino acid absorption and transport via roots. *GH_A12G0029* encodes a sulfate transporter (SULTR) that is involved in sulfate transport. *GH_A12G0282* encodes an allene oxide synthase (AOS) that is involved in JA biosynthesis. *GH_D06G2326* encodes an anthocyanidin-3-O-glucosyltransferase (UFGT) that is involved in the biosynthesis of anthocyanins.

**Table 1:** Functional annotations of candidate genes.

Gene ID	Gene name	Homologous genes in *Arabidopsis thaliana*	Functional annotation
GH_A10G1801	LEA	AT4G15910	Maintains the osmotic pressure of the cell and protects the cell membrane structure
GH_A01G0048	KCS	AT1G19440	Very-long-chain fatty acid biosynthetic process
GH_A01G2352	GST	AT5G02790	Reactive oxygen species scavenging
GH_A03G1732	NAC	AT5G13180	Response to salt stress
GH_A05G2081	MADS	AT1G22130	Response to adversity stress
GH_A05G4235	CHI	AT5G05270	Flavonoid biosynthetic process
GH_A07G2044	UGT82A1	AT3G22250	Catalyzes the glycosylation of flavonoid compounds
GH_A09G0143	CML	AT5G39670	Ca^2+^ signal effector identification
GH_A09G0432	LHT	AT5G40780	Absorption and transport of amino acids by roots
GH_A12G0029	SULTR	AT3G51895	Sulfate transport
GH_A12G0282	AOS	AT5G42650	JA biosynthesis
GH_A13G2571	MYB	AT3G46130	Response to adversity stress
GH_D02G1891	NAC	AT5G13180	Response to adversity stress
GH_D05G2419	CHS	AT5G13930	Flavonoid biosynthetic process
GH_D06G2326	UFGTs	AT4G27570	Biosynthesis of anthocyanins

The relationships between these 15 candidate genes and salt tolerance in cotton was further explored using qRT–PCR to analyze their expression patterns in cotton under salt stress at different time points (**Figure 9d**). Among these genes, *GH_A01G0048*, *GH_A03G1732*, *GH_A05G2081*, *GH_A05G4235*, *GH_A07G2044*, *GH_A09G0143*, *GH_A09G0432*, *GH_A10G1801*, *GH_A12G0282*, *GH_A13G2571*, *GH_D05G2419* and *GH_D06G2326* presented significant increases in expression under salt stress. Three genes (*GH_A01G2352*, *GH_A12G0029* and *GH_D02G1891*) presented significant decreases in expression. In summary, 15 candidate genes associated with cotton salt tolerance, including 4 TFs, were identified through WGCNA and qRT–PCR.

## 4 Discussion

The global area of saline–alkaline land is approximately 1 billion hm, and it is increasing at a rate of 1.0 × 10⁶ to 1.5 × 10⁶ hm annually. Saline–alkaline land is primarily distributed in desert and semidesert regions, but it is also common in fertile alluvial plains, river basins, coastal areas, and irrigation zones (Liu et al., 2025b). Given the limited amount of arable land, the development and utilization of saline–alkaline land are particularly important. In most cases, alkaline stress causes more severe damage to plants than does salt stress. This difference is because the hydrolysis of CO_3_^3^ and HCO_3_^3^ increases the soil pH, altering its physicochemical properties and structure and decreasing soil aeration and water conductivity, leading to a deficiency of soluble minerals and ultimately impairing root growth and survival (Bai et al., 2025). Cotton, a pioneer crop for saline–alkaline land, has been reported to be affected by alkaline stress. Under Na₂CO_3_ stress, cotton roots exhibit severe wilting and blackening, leaves lose their luster, leaf veins darken, and the chlorophyll content and relative water content decrease significantly (Zhang et al., 2018). Under NaHCO_3_ stress, the leaves wilt and lose water, the roots turn yellow, the bases of the stems turn reddish-brown, and the veins of true leaves yellow (Fan et al., 2021). However, in-depth studies on the molecular mechanisms underlying the response of cotton to alkaline stress are lacking. Therefore, investigating the regulatory mechanisms involved in the response of cotton to alkaline stress is highly important. In this study, RNA-seq and metabolomic sequencing were conducted to analyze gene expression and metabolic changes in cotton under alkaline stress at different time points. The clustering analysis and PCA of the RNA-seq and metabolomic data revealed that as the duration of alkaline stress increased, the correlations between samples decreased, whereas the number of DEGs and DAMs increased. This result could be due to the stimulation of alkaline tolerance-related gene expression in cotton, leading to the activation of more biological processes to increase the ability of the plant to adapt to alkaline stress.

Flavonoids are a group of plant secondary metabolites widely distributed in the plant kingdom that accumulate in plants in response to various abiotic stresses (such as drought, high temperature, phosphorus deficiency, and salinity) (Liu et al., 2021). The regulation of flavonoid biosynthesis has been recognized as an important mechanism by which plants resist alkaline stress, and this mechanism has been reported in crop species such as *Arabidopsis*, rice, and maize (Han et al., 2023; Li et al., 2022; M. Zhang et al., 2023). Additionally, flavonoids have antioxidant properties that allow them to scavenge ROS in response to both biotic and abiotic stresses. Studies have shown that flavonoids increase plant alkali tolerance by influencing antioxidant enzyme activity. Severe alkaline stress often inactivates antioxidant enzymes, while flavonoid accumulation effectively clears ROS (Zhu et al., 2024). Genes such as CHI, C4H, 4CL, DFR, and CHS are key components of the flavonoid biosynthesis pathway (Liu et al., 2021). In *Medicago sativa*, *MsFLS13* promotes salt–alkaline stress tolerance by increasing flavonol accumulation, the antioxidant capacity, osmotic balance, and photosynthetic efficiency (Zhang et al., 2023). In soybean, the overexpression of *GmCHI4A* and *GmCHI4B* significantly increases the total isoflavonoid content and improves salt tolerance (Zhang et al., 2024). The overexpression of *CHS1* from *Iris halophila Pall.* in transgenic *Arabidopsis* leads to reduced membrane lipid peroxidation, an increased proline content, increased antioxidant enzyme activity, and increased levels of flavonoids and other phenylpropanoid compounds, which improve salt tolerance (Liu et al., 2025c). In our transcriptomic analysis, we also identified 4 C4H genes (*GH_A10G1944*, *GH_A13G2635*, *GH_D10G2039* and *GH_D13G2625*), 10 CHI genes (*GH_A02G1972*, *GH_A05G4235*, *GH_A11G1837*, *GH_A12G0599*, *GH_A13G0216*, *GH_D03G0093*, *GH_D04G0140*, *GH_D11G1872*, *GH_D12G0618* and *GH_D13G0214*), and 7 CHS genes (*GH_A02G0270*, *GH_A05G3278*, *GH_A10G2687*, *GH_D02G0295*, *GH_D05G2419* and *GH_D10G1568*) that were upregulated in cotton under alkaline stress. Furthermore, our metabolomic data revealed a regulatory network of flavonoid biosynthesis genes and metabolites in cotton under alkaline stress. Notably, *GH_D11G1872* (CHI) expression was significantly negatively correlated with peonidin 3-(6’’-p-coumarylglucoside), delphinidin 3-O-beta-D-sambubioside, and malvidin 3-(6’’-p-coumarylglucoside) levels. These comprehensive findings suggest that cotton can increase flavonoid accumulation by upregulating key genes in the flavonoid biosynthesis pathway, thereby effectively scavenging ROS and increasing its survival rate under alkaline stress.

Plants trigger changes in the levels of endogenous hormones (such as JA and abscisic acid (ABA)) to respond to alkaline stress (Chen et al., 2024; Liu et al., 2022; Yan et al., 2019). JA plays a crucial role in the response to abiotic stresses such as alkalinity. For example, the exogenous application of JA significantly reduces the Na^+^ content in rice and alleviates salt stress-induced damage and photosynthetic impairment in seedlings (Hussain et al., 2022). Studies also indicate that the expression of JA signaling pathway genes in significantly induced in *Arabidopsis thaliana* under alkaline stress (Pérez-Martín et al., 2021). Furthermore, a previous study showed that a JA pretreatment can increase the alkaline stress tolerance of corn by altering ion homeostasis and the activities of antioxidant and glyoxalase systems (Mir et al., 2018). In *Arabidopsis thaliana*, *AtJAZ10* expression decreases in response to alkaline stress, and the corresponding T-DNA insertion mutant shows significantly better growth under alkaline conditions than wild-type plants (Wang et al., 2020). Recent research on tomato has revealed that the *SlWRKY42*–*SlMYC2* module regulates JA signaling, lowers the Na^+^/K^+^ ratio, and thus enhances salt–alkaline stress tolerance (Liu et al., 2025c). In this study, through RNA-seq and metabolomic analyses of cotton under alkaline stress, we observed the significant enrichment of the alpha-linolenic acid metabolism pathway. This pathway, which is an important route for JA biosynthesis, led us to construct a regulatory network between JA biosynthetic genes and metabolites. We identified the rate-limiting enzymes AOC (*GH_D08G0423*) and AOS (*GH_A04G1388*, *GH_A12G0282* and *GH_D06G0112*), as well as increased JA levels, in cotton under alkaline stress. Through WGCNA, we also identified *GH_A12G0282* (AOS) as an important candidate gene for alkali tolerance in cotton. These findings provide new insights for future research into the role of JA in the alkali tolerance of cotton. However, when the impact of JA on the alkali tolerance of cotton is studied, the interconnections between various plant hormones are crucial. Therefore, a focus on the interactions between two specific plant hormones and a consideration of the collective actions of multiple plant hormones on plant stress resistance are important.

High-pH stress disrupts the root proton gradient across the membrane, inhibiting Na^+^ efflux, which leads to Na^+^ overaccumulation. This phenomenon has been validated in various plant species and serves as a critical physiological basis for alkaline stress-induced damage (Lu et al., 2025). Ca^2+^ is considered one of the most important signaling molecules involved in the response to salt stress, and elevated Ca^2+^ levels can alleviate Na+ toxicity. Alkaline soils typically contain large amounts of CO_3_^2-^, which directly precipitates Ca^2+^, leading to a dramatic decrease in the activity and availability of Ca^2+^ around the roots and resulting in a severe deficiency of bioavailable Ca^2+^ (Hao et al., 2024). Indeed, under alkaline stress, many Ca^2+^-related genes in cotton exhibit significant changes in expression, such as the upregulation of the CBL and CIPK genes. Additionally, we identified *GH_A09G0143*, which encodes a CML protein that primarily participates in Ca^2+^ signal effector recognition. Studies have reported that a CML family transmembrane protein can prevent excess ROS accumulation in roots and potentially regulate Ca^2+^ signaling, vesicular transport, and the formation of diffusion barriers, thereby improving plant tolerance to salt stress (Zhang et al., 2021).

Many studies have shown that transcription factors can directly or indirectly regulate the expression of genes related to alkaline stress responses, thereby affecting the ability of plants to adapt to alkaline stress (Yang et al., 2024). In recent years, significant progress has been made in the research of transcription factors involved in plant alkali tolerance, with dozens of transcription factors identified as participating in plant responses to alkaline stress (Fang et al., 2021). These transcription factors belong to several major families, such as AP2/ERF, bZIP, NAC, MYB, MYC and WRKY (Erpen et al., 2018). We also identified 579 differentially expressed TFs among the 6610 DEGs, with the highest proportions observed for AP2/ERF, MYB, bHLH, WRKY, NAC, bZIP, C2H2, HD-ZIP, and GRAS transcription factors. For example, *BpMYB06* mainly regulates the response of birch to alkaline stress by increasing reactive oxygen species scavenging and regulating the osmotic and ion balance, thus affecting the stomatal aperture (Zhou et al., 2025). *MYB5* coordinates the biosynthetic regulation of sesquiterpenes and proanthocyanidins in rose in response to alkaline stress by directly regulating the expression of *TPS31* and *ANR* (Wang et al., 2024a). QTL-seq and QTL mapping revealed that *OsMYB305* can regulate the alkali tolerance of rice, with further studies indicating that *OsMYB305* modulates the alkali tolerance of rice by affecting the transport of Na^+^ and K^+^ in the root system and in seedlings (Li et al., 2024). Through WGCNA combined with qRT–PCR, *GH_A13G2571* (MYB) was identified as a candidate gene for alkali tolerance in upland cotton, with the peak expression occurring at 8 hours of alkaline stress (fold change > 5). By regulating the expression of NAC transcription factors, the morphology and growth of plant roots can be influenced to respond to stress tolerance. *GmNAC06* can control the Na^+^/K^+^ ratio in root hairs, inducing the expression of genes related to proline, glycine betaine, and ROS metabolism, thereby lowering the osmotic potential and ROS levels in soybean plants under salt stress and maintaining ionic homeostasis (Li et al., 2021). Transgenic lines overexpressing sorghum *GsNAC2* presented significant increases in the plant height, dry weight, water content, root vitality, leaf length, chlorophyll content, stomatal conductance, relative root vitality, relative chlorophyll content, relative stomatal conductance, and relative transpiration rate (Wu et al., 2023). We also identified two NAC transcription factors (*GH_A03G1732* and *GH_D02G1891*), among which *GH_A03G1732* showed peak expression at 4 h of alkaline stress (fold change > 2). In addition to these genes, we identified numerous TFs involved in the alkaline stress response. These genes and TFs are important genetic resources for understanding the alkali tolerance of cotton and could serve as key targets for future research.

## 5 Conclusions

This study combined RNA-seq and metabolomics to explore the molecular mechanisms involved in the response of cotton to alkaline stress at different time points. The KEGG enrichment analysis revealed that the flavonoid biosynthesis and JA biosynthesis pathways were significantly enriched in cotton under alkaline stress, and the expression levels of several key genes changed significantly in response to stress. In particular, genes involved in flavonoid biosynthesis, such as C4H, CHI, and CHS, were upregulated by alkaline stress, promoting flavonoid accumulation and increasing plant stress tolerance. By constructing a regulatory network between flavonoid biosynthesis genes and metabolites, we determined that *GH_D11G1872* (CHI) expression is significantly correlated with the levels of several flavonoid metabolites, potentially playing a crucial role in the response to alkaline stress. In terms of JA biosynthesis, the expression of the AOC and AOS genes, as well as the accumulation of JA and methyl dihydrojasmonate, increased significantly in plants under alkaline stress, highlighting the important role of JA in the alkali tolerance of cotton. WGCNA identified key candidate genes closely related to the alkaline stress response, with *GH_A12G0282* (AOS) emerging as an important candidate gene that may play a crucial role in the alkali tolerance of cotton. Furthermore, genes related to Ca^2+^ signaling, such as CBL, CIPK, and CML, were upregulated in response to alkaline stress, potentially increasing cotton tolerance to alkaline stress by regulating Ca^2+^ homeostasis and ROS clearance. Overall, this study provides an in-depth investigation into the molecular mechanisms involved in the response of cotton to alkaline stress through multiomics analyses and identifies key genes and metabolites. These findings lay a theoretical foundation for the future molecular breeding of alkali-resistant cotton.

## Data Availability

The RNA-seq data presented in the study are deposited in the NCBI repository under accession number PRJNA1234302.

## Supplementary material

Supplementary Figure 1 PCA of metabolomic samples.
