# Supplementary figures and images for: Integrated transcriptomic and metabolomic analyses provide new insights into alkaline stress tolerance in Gossypium hirsutum
Source: Front Plant Sci. 2025 Jun 3;16:1604606. doi: 10.3389/fpls.2025.1604606 (PMC12170613; doi:10.3389/fpls.2025.1604606)

PC2 (15.7%)

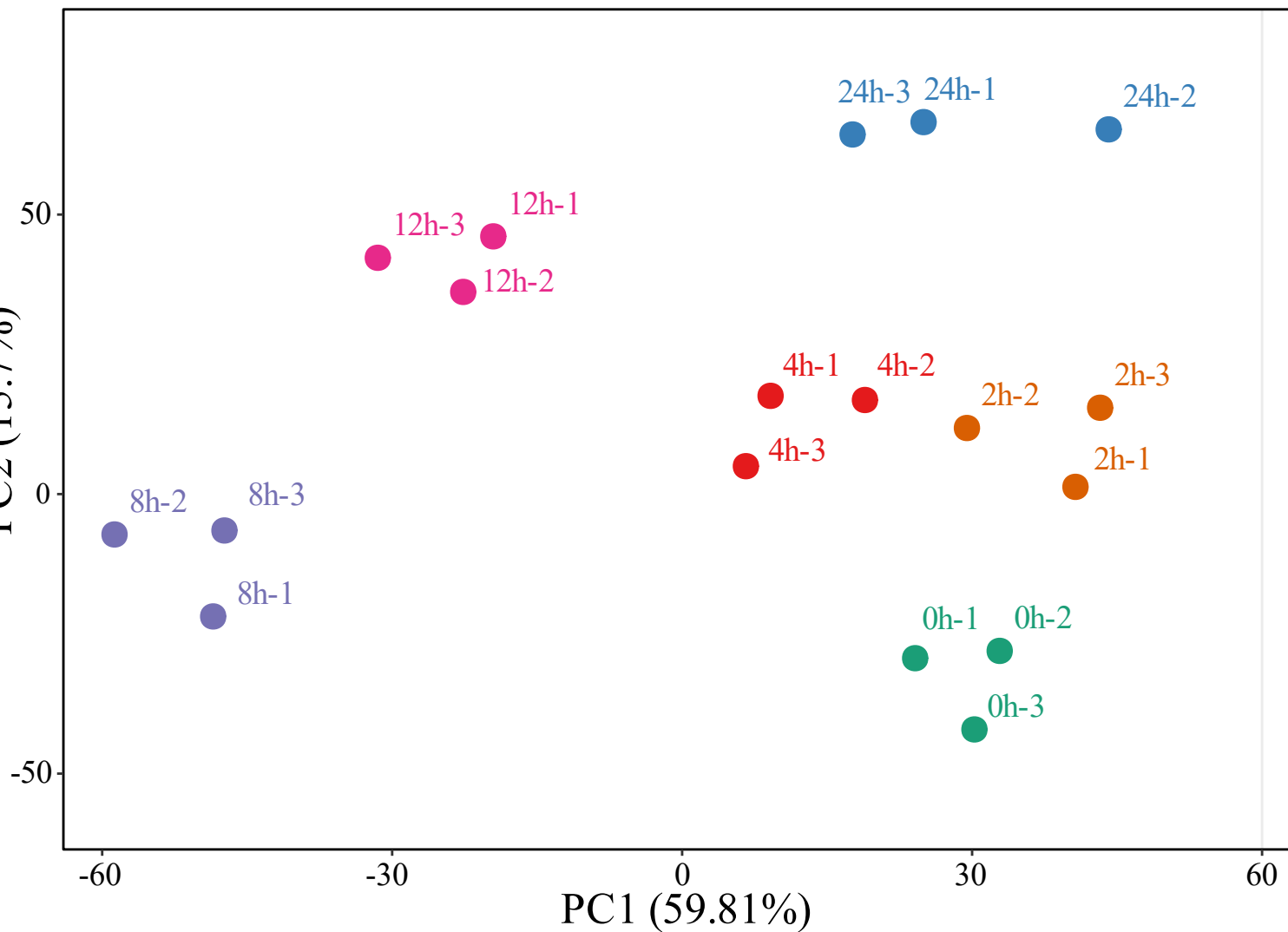

PC1 (59.81%)

Supplement: Supplementary Figure 1 — PCA of metabolomic samples. [file DataSheet1.pdf]
